# Per-oral interstitial brachytherapy catheter insertion for boost in case of recurrent tonsillar carcinoma: dosimetry and clinical outcome

**DOI:** 10.1259/bjrcr.20190059

**Published:** 2020-02-12

**Authors:** Naoya Murakami, Seiichi Yoshimoto, Satoshi Nakamura, Masakazu Uematsu, Tairo Kashihara, Kana Takahashi, Koji Inaba, Kae Okuma, Hiroshi Igaki, Yuko Nakayama, Jun Itami

**Affiliations:** 1 Department of Radiation Oncology, National Cancer Center Hospital, Tokyo, Japan; 2 Department of Head and Neck Surgery, National Cancer Center Hospital, Tokyo, Japan

## Abstract

High-dose-rate interstitial brachytherapy (HDR-ISBT) is relatively rarely applied for the head and neck cancer. However, its dose distribution is more confined than intensity modulated radiation therapy (IMRT) and can deliver higher dose while sparing surrounding normal tissues. In this case report, the effectiveness of HDR-ISBT as a boost following IMRT for post-operative recurrent oropharyngeal cancer patient was indicated. A 73-year-old male who developed local recurrence after surgery for oropharyngeal squamous cell carcinoma. Salvage IMRT up to 70 Gy concurrent with weekly cetuximab was planned. However, CT taken at 60 Gy found a residual tumor, then, boost HDR-ISBT was proposed. 1 week after 60 Gy of IMRT, HDR-ISBT, 12 Gy in 2 fractions, was delivered under local anesthesia. MRI taken 2 months after HDR-ISBT showed no residual tumor. It was demonstrated that boost HDR-ISBT following IMRT for local recurrence of oropharyngeal cancer was performed safely and showed favorable efficacy.

## Introduction

Because it is difficult to safely insert interstitial applicators in the head and neck region, brachytherapy is rarely used in head and neck region except early stage tongue cancer,^1–5^ superficial oral cavity cancer,^6–9^ or nasopharyngeal cancer.^10–12^ External beam radiation therapy plays an important role in the management of head and neck cancer either in the form of definitive treatment,^13–15^ post-operative adjuvant treatment,^16–18^ or salvage treatment.^19,20^ It was found that concurrent administration of chemotherapy with radiation therapy increases the possibility of tumor control.^16–18^ Moreover, since the introduction of intensity modulated radiation therapy (IMRT), it has been possible to deliver tumoricidal dose to the clinical target while sparing high dose to surrounding normal tissues.^21,22^ However, despite improved radiation therapy in the head and neck cancer, tumor resistance against radiation can be frequently encountered in daily clinical practice.

Although early stage tongue cancers are relatively frequently treated by interstitial brachytherapy (ISBT),^1–5,23,24^ its application in the head and neck region is nowadays not frequent because complicated anatomical structures of the head and neck region preclude brachytherapist to insert interstitial needles safely. After the adequate training and experience, however, ISBT can play an important role not only for primary tumors,^25^ but also in the recurrent tumors^26–29^ in the management of head and neck malignancies. In this case report, the authors successfully utilized high-dose-rate ISBT (HDR-ISBT) as a boost for local recurrent tumor after primary surgery for oropharyngeal squamous cell carcinoma patient. Written informed consent was obtained from the patients and this case report was approved by the Institutional Review Board of National Cancer Center Hospital (approved number is 2017-331) according to the ethical standards laid down in the Declaration of Helsinki.

## Clinical presentation

**Figure 1.**
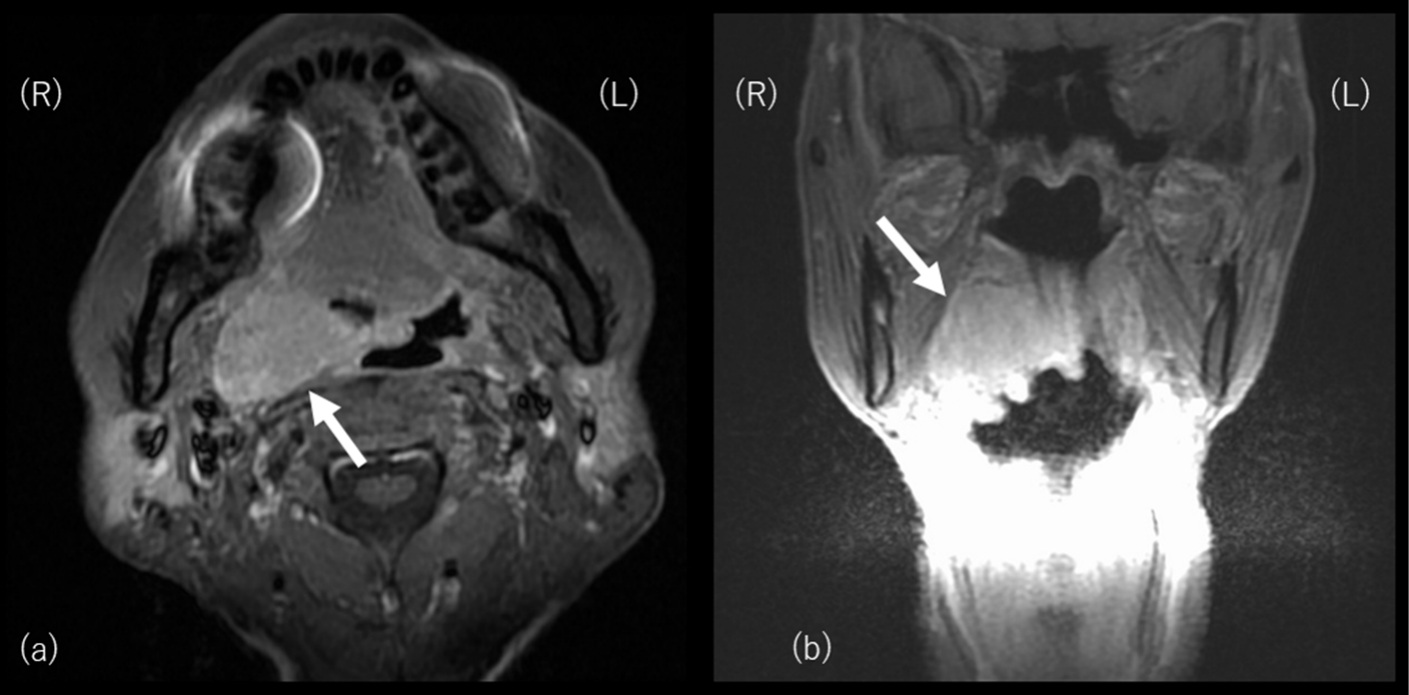
MR images of oropharyngeal squamous cell carcinoma in the right tonsil before primary surgery. Figure 1 (a) and (b) shows an axial and sagittal image of the primary tumor (white arrow).

A 73-year-old-male, who had 17 pack-year smoking history, received primary tumor resection with the pull-through method and ipsilateral selective conservative neck lymph node dissection (Level IIa and III) followed by the right anterolateral thigh (ALT) flap reconstruction for clinical T3N0, p16 positive, squamous cell carcinoma in the right tonsil (Figure 1). Pathologically, the surgical margin was negative and no positive metastatic neck lymph node was found, therefore, no adjuvant radiation therapy was administered.

**Figure 2.**
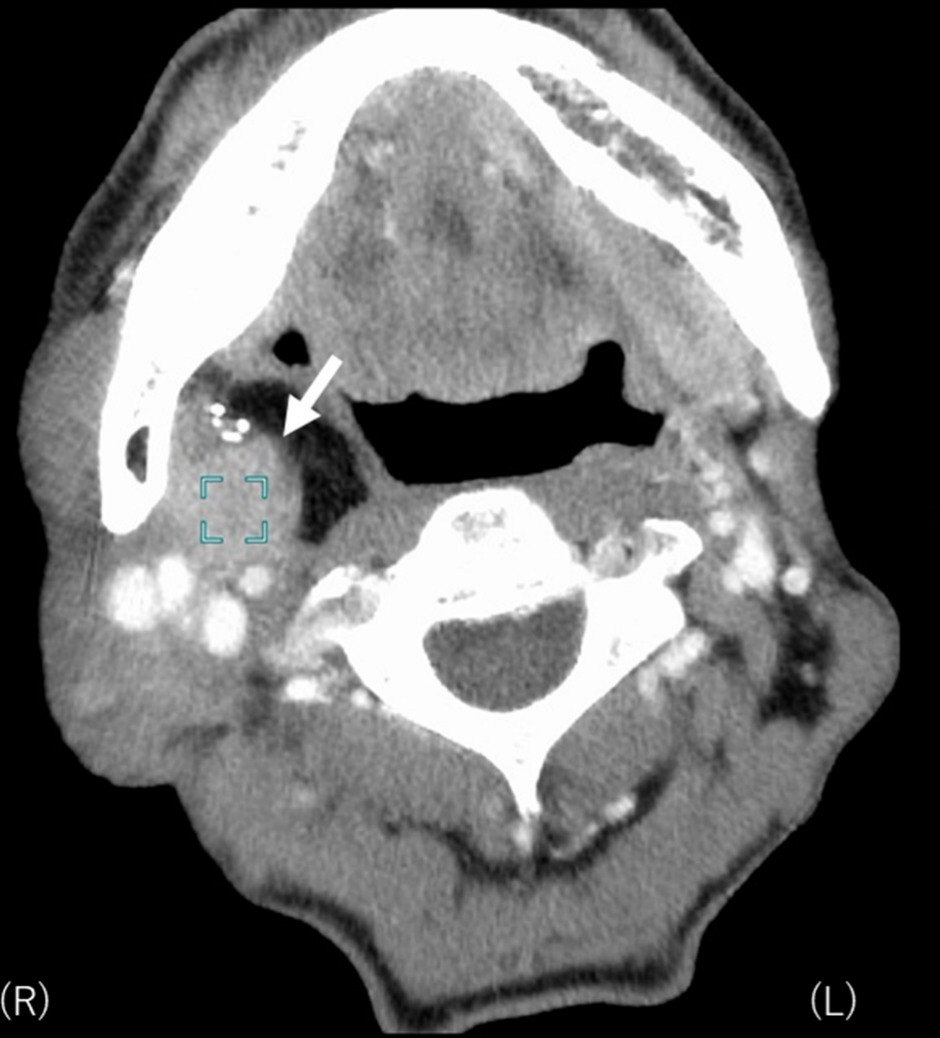
CT of the recurrent tumor beneath the ALT flap in the right side of the tonsillar area (white arrow) touching the right internal carotid artery. ALT, anterolateral thigh.

**Figure 3.**
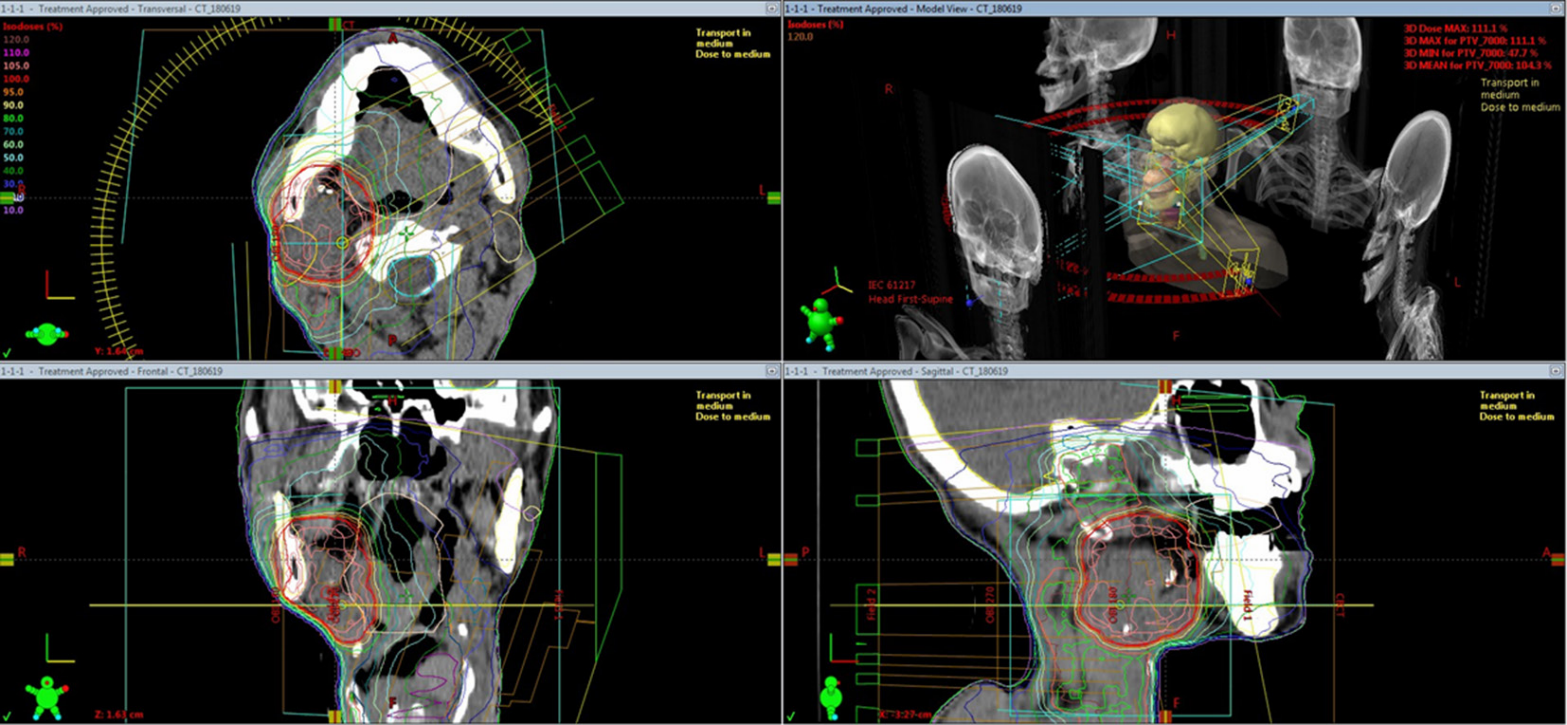
Figure shows dose distribution of intensity modulated radiation therapy with simultaneous integrated technique. The recurrent tumor was covered with the red isodose line while prophylactic right neck lymph node area was covered by the green isodose line.

**Figure 4.**
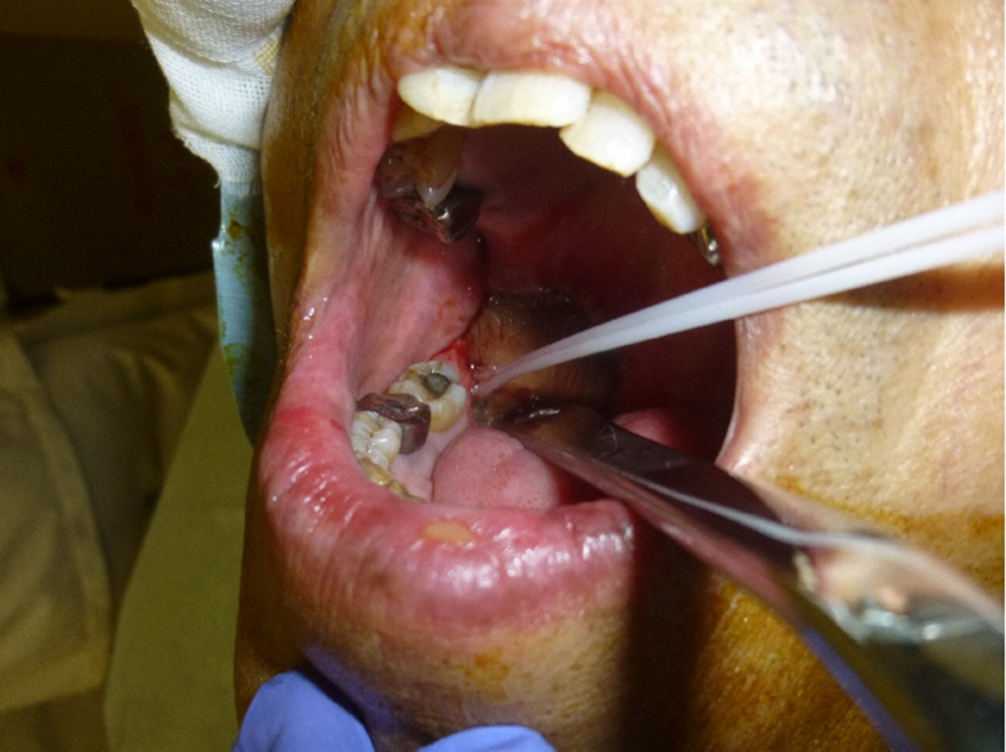
Under local anesthesia and sedation, two 5 French ProGUide^®^ plastic needles (Nucletron BV, Veenendaal, The Netherlands) were inserted transorally through the ALT flap. Depth of the needles were determined by CT image. ALT, anterolateral thigh.

**Figure 5.**
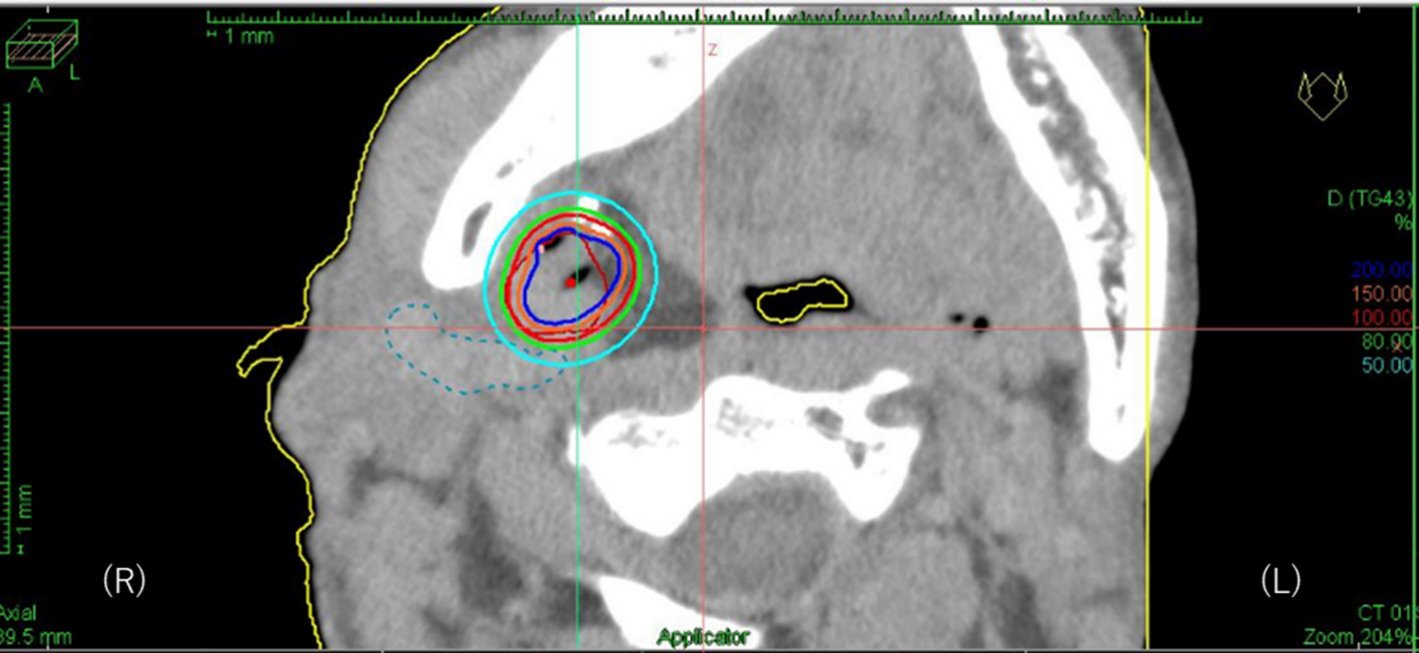
Isodose distribution of the interstitial implant with the red and blue line representing the 100 and 200% isodose, respectively. The dotted light blue line represents vessels.

**Figure 6.**
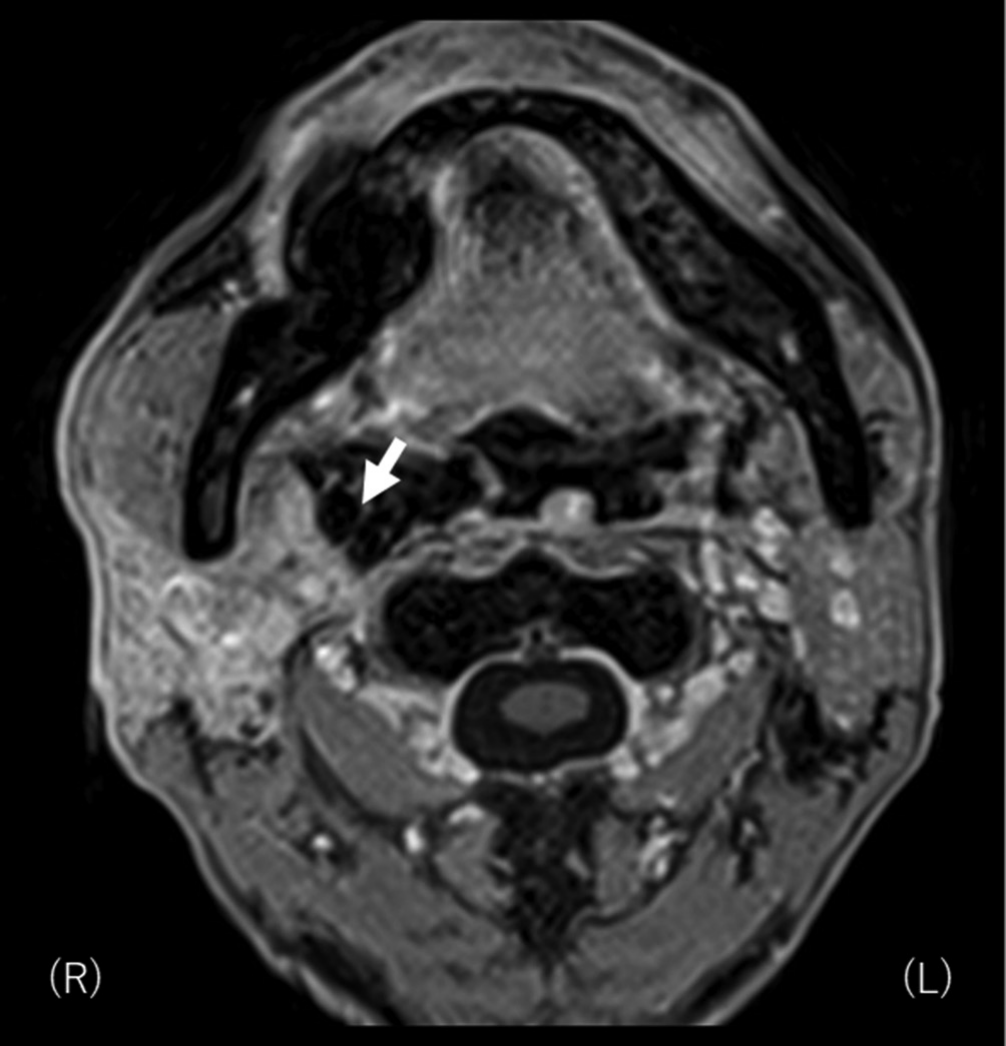
MR image was taken 2 months after interstitial brachytherapy. No evident residual tumor was found beneath the ALT flap. ALT, anterolateral thigh.

18 months later after surgery, follow-up CT found an isolated local recurrence just behind the ALT flap in the primary lesion (Figure 2). Because the recurrent tumor touched internal carotid artery and repeated reconstruction surgery was considered to be relatively difficult, salvage surgery was not performed. As this patient had mild kidney dysfunction and he wanted outpatient-based treatment while working as normal as possible, salvage concurrent chemoradiation with cisplatin which requires hospitalization and hydration was not selected but salvage radiation therapy with weekly cetuximab was planned.^30^ Dose distribution of IMRT for the recurrent tumor is shown in Figure 3. Initially, the prescribed total dose of IMRT was set to be 70 Gy in 35 fractions in conventional fractionation with IMRT plan being normalized so that the 95% of the planning target volume must receive larger than the prescribed dose (D95). However, CT taken at 60 Gy found still evident residual tumor and boost image-guided HDR-ISBT was recommended because transoral brachytherapy applicator insertion through the ALT flap was considered to be easy. After obtaining the patient’s consent, IMRT was stopped at 60 Gy and 2 sessions of HDR-ISBT, 12 Gy in 2 fractions, 1 fraction per day in consecutive 2 days, was performed a week after IMRT in outpatient setting. Because it was supposed that needle fixation overnight was difficult, needles were removed after the first irradiation and they were inserted again before the second irradiation. Under local anesthesia and sedation, two 5 French ProGUide^®^ plastic needles (Nucletron BV, Veenendaal, The Netherlands) were inserted transorally through the flap (Figure 4). The recurrent tumor could be easily palpated through oral cavity under the reconstructed flap, therefore, initially the needles were inserted by the finger guidance. Then, the depth of the needles were determined after obtaining the CT image. Because the recurrent tumor was just next to the carotid artery and retromandibular vein, CT with contrast enhancement (Oiparomin 370; Fuji Pharmaceutical Company, Toyama, Japan) was taken and depth of the needle was determined. Dose calculation was performed using Oncentra Brachy v. 4.5.1 (Nucletron, an ELEKTA company, ELEKTA AB, Stockholm, Sweden) so that 100% isodose line covered the CTV and CTV-D90 became larger than the prescribed dose based on CT image (image-guided brachytherapy) (Figure 5). As for HDR-ISBT, dose non-uniformity ratio (*DNR*)^31^ and conformal index (*COIN*)^32^ were calculated for gross tumor volume (GTV) at the time of brachytherapy according to the following equations.

$$DNR=V_{150}/V_{100}$$

where *V_100_* and *V_150_* are the absolute volumes in ml irradiated by 100 and 150% of the prescribed dose (6 Gy), respectively.

$$COIN=GTV_{ref}/V_{GTV}•GTV_{ref}/V_{ref}$$

where *GTV_ref_* is the absolute volume of the GTV irradiated by the prescribed dose, *V_GTV_* is the absolute volume of GTV, and *V_ref_* is the volume irradiated by the prescribed dose. Mean value of DNR and COIN for two HDR-ISBT sessions were 0.60 and 0.42, respectively. Rigid image registration between CT images for external beam radiation therapy (EBRT) and HDR-ISBT was performed where mandible and surgical clips located adjacent to the recurrent tumor by chance were used as reference using the Mim Maestro registration software (Mim Maestro v. 6.8.5., MIM software Inc, Cleveland). After summation of dose contribution from EBRT and HDR-ISBT, GTV D_90_, mandible D_2cc_, mandible V_70Gy_, carotid artery D_0.5cc_ (α/β = 3 Gy, EQD_2_) was found to be 82.9 Gy (α/β = 10 Gy, EQD_2_), 67.8 Gy, 0.33 ml, and 63.4 Gy (α/β = 3 Gy, EQD_2_), respectively. No severe acute toxicity was noted with regard to applicator insertion. MRI taken 2 months after HDR-ISBT (Figure 6) showed no residual tumor without any palpable nodule beneath the reconstructed flap with again no late severe toxicity, including late osteonecrosis of the jaw.

## Discussion

Standard therapy for patients with isolated local failure is salvage surgery. However, because the recurrent tumor was close to the carotid artery, the recurrent tumor was considered to be inoperable and radiation therapy was offered. Because p16 status of this patient was positive, the response against cetuximab-radiation therapy was considered to be favorable, therefore, IMRT was selected as a salvage modality of treatment. However, since it was found that evident residual tumor still existed after 60 Gy of IMRT, IMRT was stopped and image-guided HDR-ISBT was offered as a boost. In Radiation Therapy Oncology Group-0129 trial, recursive partitioning analysis identified low-, intermediate-, and high-risk group based on HPV status, tobacco pack-years, tumor stage, and nodal stage.^33^ More than 10 pack-years was regarded as a risk factor in this study and our patient had this unfavorable factor which could be a reason why he responded poorly against cetuximab-radiation therapy; although the risk classification for primary oropharyngeal cancer is not for recurrent tumors, it is not applicable for our patient.

While early stage tongue cancer are relatively frequently treated by interstitial brachytherapy,^1–5,23,24^ application of brachytherapy in the head and neck region in general became nowadays relatively not frequent. However, even after the introduction of IMRT which enable radiation oncologist to deliver tumoricidal dose to the target while sparing normal tissues surrounding the tumor, if interstitial needles can be inserted safely, the dose distribution of brachytherapy is more confined and can deliver a higher dose while sparing surrounding normal tissues than IMRT: unlike EBRT, inhomogeneity is a specific feature for brachytherapy. As shown in the results, mean value of DNR and COIN for two HDR-ISBT sessions were 0.60 and 0.42, respectively. Delivering over 80 Gy to the GTV with this high conformity is only possible with brachytherapy. Because of anatomic complexity in the head and neck region, it naturally requires adequate training and experience for safely insert interstitial needles. Pernort et al and Levendag et al reported a large cohort of oropharyngeal cancer patients treated by ISBT.^34,35^ Tselis et al utilized ISBT for neck lymph node metastasis in the salvage setting.^28,29^ Those brachytherapists showed the effectiveness of ISBT in the head and neck region. Similar to the success story of the image-guided gynecologic brachytherapy,^36^ with the help of image guidance, it is possible to insert needles in the complicated anatomical sites such as head and neck. In the future, possibly with the assistance of robotic technology, it would be easier to insert interstitial needles while avoiding critical anatomical structures such as artery, vein, or nerves and renaissance of brachytherapy in the head and neck would happen.

There was a limitation to this case report. In the initial plans, ISBT boost was not intended to be used for this case and the recurrent tumor was initially handled only with 70 Gy of IMRT. Dose per fraction used in this case was relatively higher than recommended boost ISBT dose of 3.5–4 Gy per fraction,^23,24^ therefore, further observation should be needed to see the safety of this combination treatment.

In this case report, it was demonstrated that adding image-guided HDR-ISBT as a boost following IMRT was a very effective way of treating recurrent head and neck patient. When applicable, a radiation oncologist should always take into account of adding boost HDR-ISBT for a poor responder to conventional IMRT for head and neck cancer patients.

## Learning points

Brachytherapy for oropharyngeal cancer is currently rarely performed. However, if used properly, similar to brachytherapy for other organs, it was shown that interstitial brachytherapy was also a very effective local treatment in head and neck tumors.
